# Diagnostic Yield and Safety of Medical Thoracoscopic Biopsy in Undiagnosed Exudative Pleural Effusion: A Five-Year Retrospective Study From a Tertiary Care Center in South India

**DOI:** 10.7759/cureus.102384

**Published:** 2026-01-27

**Authors:** John Sonia Kallarakal, Venugopal K.P.

**Affiliations:** 1 Pulmonary Medicine, Government Medical College, Kottayam, Kottayam, IND

**Keywords:** complications, diagnostic yield, malignancy, medical thoracoscopy, pleural effusion, tuberculosis

## Abstract

Background: Medical thoracoscopy is a minimally invasive procedure with high diagnostic yield in undiagnosed exudative pleural effusions. There is limited data on its efficacy and safety from high-volume tertiary centers in India, considering the changing etiological spectrum.

Methods: This is a retrospective observational study including all consecutive patients who underwent medical thoracoscopy for undiagnosed exudative pleural effusion at a tertiary care teaching hospital in South India over a five-year period. Data were obtained from departmental records and analyzed for diagnostic yield, etiology, macroscopic findings, therapeutic interventions, complications, and outcomes.

Results: A total of 305 patients were included (mean age 57 ± 15.5 years; 60.3% male). An etiological diagnosis was achieved in 303 patients, yielding a diagnostic yield of 99.4%. Malignancy was the most common diagnosis (64.6%), predominantly lung adenocarcinoma (77.1% of malignancies), followed by benign causes (35.4%), with tuberculosis accounting for 14.4% overall. Nodules (59.7%) and adhesions (41.3%) were the most frequent macroscopic findings, correlating strongly with malignant and infective etiologies, respectively. Talc pleurodesis and adhesiolysis were performed in 18.7% and 23.0% of cases, respectively. Complications were predominantly minor: post-procedure pain (66.6%), followed by subcutaneous emphysema (7.2%). Major complications were rare (hemorrhage 0.3%, prolonged air leak <2%). No procedure-related mortality occurred.

Conclusion: Pulmonologist-performed medical thoracoscopy demonstrated high diagnostic yield and an excellent safety profile in undiagnosed exudative pleural effusions. The predominance of malignancy reflects changing epidemiological trends and supports early thoracoscopic evaluation in undiagnosed exudative pleural effusion.

## Introduction

Medical thoracoscopy was first introduced by Hans-Christian Jacobaeus in 1910 for the examination of the pleural space [1]. Medical thoracoscopy is a minimally invasive procedure performed by pulmonologists under local anesthesia and conscious sedation. Unlike video-assisted thoracoscopic surgery (VATS), which requires general anesthesia and multiple ports, medical thoracoscopy uses a single-entry point, making it less invasive and more cost-effective [2,3]. It is primarily a diagnostic procedure but is also used for therapeutic purposes, such as talc pleurodesis for malignant pleural effusions or recurrent pneumothorax, adhesiolysis in empyema, and drainage of loculated effusions [4,5].

The etiological diagnosis of pleural effusion presents a considerable challenge; 20-40% of cases remain undiagnosed after initial thoracentesis, pleural fluid analysis, and closed pleural biopsy [6,7]. Medical thoracoscopy enables direct visualization of the pleural cavity and targeted biopsies of the parietal pleura, large enough for histopathological and immunohistochemical analysis [8]. Recent meta-analyses and guidelines confirm its high diagnostic sensitivity, exceeding 90-95% for malignant pleural effusions and approaching 100% for tuberculous effusions in endemic regions [9-11].

Medical thoracoscopy is a relatively safe procedure in experienced hands with mortality rates below 0.5%. Major complications (e.g., hemorrhage, bronchopleural fistula, pneumothorax, pneumonia, or port-site tumor seeding) are seen in 1-5% of cases [12,13]. Minor complications, including subcutaneous emphysema, minor bleeding, site infection, fever, or atrial fibrillation, are more common but self-limiting [14]. The British Thoracic Society and American Thoracic Society guidelines endorse the role of medical thoracoscopic biopsy for undiagnosed exudative effusions in both diagnosis and management of pleural diseases [15-17].

In a retrospective observational study regarding all consecutive medical thoracoscopies performed with diagnostic intent over 30 years, the overall diagnostic yield was 71%, increasing from 57% to 79%. The most common diagnosis was malignancy; tuberculosis was the most common benign cause. It was concluded that accurate patient selection is vital for achieving these results [18].

In a retrospective study of 407 patients with exudative pleural effusions, medical thoracoscopy was a safe procedure. The most common cause was tuberculosis, accounting for 84.5% of all cases. The diagnostic yield of medical thoracoscopy for tuberculous pleural effusion was 91.4%. Malignant pleural effusions accounted for 5.2% of cases. Minor bleeding occurred in 1.2% of cases, with no procedure-related mortality observed [19].

In a four-year experience on medical thoracoscopy, the most typical complication was post-procedural pain, followed by subcutaneous emphysema. Overall, it is a safe procedure with no procedure-related mortality reported [20].

A nine-year study to assess the efficacy and safety of medical thoracoscopy in undiagnosed pleural effusion in a Chinese population revealed an overall diagnostic efficiency of 92.6%; the only severe complication was empyema, seen in 0.4% of patients [21].

However, data on diagnostic yield, safety, and complications in our patient population remain limited, emphasizing the need for localized evaluation.

The primary objective of the study is to determine the diagnostic yield of medical thoracoscopic biopsy in patients with exudative pleural effusions of undetermined etiology in a tertiary care setting. The following are the secondary objectives: (1) characterize the etiological spectrum of confirmed diagnosis; (2) evaluate the safety profile and incidence of immediate/peri-procedural complications; and (3) assess the correlation between initial clinical diagnosis and final histopathological diagnosis.

## Materials and methods

Study design and setting

This retrospective observational study was conducted at the Department of Pulmonary Medicine in a tertiary care teaching hospital in Kerala, India. All consecutive patients who underwent medical thoracoscopy for an undiagnosed exudative pleural effusion during the five-year study period were included.

Ethical considerations

The study was approved by the Institutional Ethics Committee of Government Medical College, Kottayam, Kerala, India (approval no. 53/2022). Permission to access data was obtained from the Head of the Department of Pulmonary Medicine and the Superintendent of the Medical Records Library.

Patient selection and data collection

Patients were eligible if they had an exudative pleural effusion (defined by Light's criteria) [7] that remained undiagnosed following initial investigations, including thoracentesis with pleural fluid analysis (biochemistry, cytology, and microbiology) and, where performed, closed pleural biopsy, after confirming no contraindications (e.g., uncorrectable coagulopathy, inability to tolerate positioning). Data were collected retrospectively from the departmental thoracoscopy register and hospital medical records using a structured proforma. The data included patient demographics (age, sex), clinical history (presenting symptoms, comorbidities, smoking status, occupational exposure), imaging findings, pre-procedure pleural fluid results, thoracoscopic macroscopic appearances, biopsy histopathology and microbiology, any therapeutic interventions performed, duration of intercostal drainage, post-procedure hospital length of stay, and complications.

Procedure

Medical thoracoscopy was performed in an interventional pulmonology suite in the Pulmonary Medicine department by experienced pulmonologists using a rigid Karl Storz thoracoscope [8]. Written informed consent was obtained before each procedure, including risks of complications like pain, subcutaneous emphysema, pneumothorax, infection, and bleeding. Patients were positioned in the lateral decubitus position with the affected side up. The procedure was conducted under local anesthesia (2% lidocaine, up to 20 mL administered in layers: skin, subcutaneous tissue, intercostal muscles, and parietal pleura) and conscious sedation (midazolam 1-5 mg total ± fentanyl 25-100 µg total), with continuous monitoring of vital signs and oxygen saturation (supplemental oxygen 2-4 L/min via nasal prongs to maintain SpO₂ > 92%), ECG, and noninvasive blood pressure monitoring.

The entry site was identified and marked using thoracic ultrasound to ensure safe access (mid-axillary line, typically 6th-8th intercostal space at the largest fluid pocket, avoiding the neuromuscular bundle and internal mammary artery) [3]. A single-port technique was employed with a 1-1.5 cm horizontal skin incision, blunt dissection through intercostal muscles, and a trocar inserted bluntly into the pleural space, with immediate fluid aspiration to confirm entry and create working space. After trocar insertion, the pleural cavity was inspected thoroughly (systematic visualization of parietal, visceral, diaphragmatic, and mediastinal surfaces; pleural fluid drained via suction). Abnormal parietal pleural areas (nodules, plaques, adhesions, and inflammation) were identified, and targeted biopsies (three to five samples) were obtained using rigid forceps. Random biopsies from normal-appearing parietal pleura were taken if abnormalities were limited. Adhesiolysis was performed if loculations were present (blunt dissection to improve visualization or drainage), and talc pleurodesis (4-5 g sterile graded talc poudrage using an applicator) was undertaken in selected cases (confirmed malignancy with recurrent pleural effusion as per patient consent). At the conclusion of the procedure, an intercostal drain (28G) was inserted through the port site and connected to an underwater seal system. Procedures adhered to principles outlined in the British Thoracic Society pleural disease guidelines [3,15].

Patients were monitored post-procedure for immediate complications, like pain and re-expansion pulmonary edema. Complications were classified as major (e.g., significant hemorrhage requiring transfusion, bronchopleural fistula, prolonged air leak >7 days, pneumothorax requiring intervention, pneumonia, empyema, wound infection) or minor (e.g., subcutaneous emphysema, minor bleeding, transient fever).

The intercostal drain was removed when output was <100-150 mL/day, no air leak was present, and a chest X-ray confirmed lung re-expansion.

Statistical analysis

Data were entered into Microsoft Excel (Microsoft Corporation, Redmond, Washington) and analyzed using IBM SPSS Statistics for Windows, Version 19 (Released 2010; IBM Corp., Armonk, New York). Categorical variables were presented as frequencies and percentages. Continuous variables were summarized as mean ± standard deviation (SD) if normally distributed or median (interquartile range) otherwise. Associations between categorical variables were assessed using the chi-square test or Fisher's exact test, as appropriate. Correlations between quantitative variables were evaluated using Pearson’s correlation coefficient. A two-sided P-value < 0.05 was considered statistically significant.

## Results

Data on 305 patients who underwent medical thoracoscopic biopsy at a tertiary care center for the diagnosis of exudative pleural effusion of undetermined etiology during the study period were obtained. The demographic details of the study subjects are presented in Tables 1-3.

**Table 1:** Age distribution of the study subjects

Age (years)	Number of subjects	Percentage (%)
≤20	8	2.6
21–40	35	11.5
41–60	126	41.3
61–80	125	41
>80	11	3.6

**Table 2:** Sex distribution of the study subjects

Sex of the study subjects	Number of subjects	Percentage (%)
Males	184	60.3
Females	121	39.7

**Table 3:** Distribution of study subjects based on smoking status

Smoking status	Number of subjects	Percentage (%)
Nonsmoker	161	52.8
Reformed smoker	97	31.8
Current smoker	47	15.4

The mean age of the study population was 57 years, with an SD of 15.5. The age distribution showed a predominance in the middle-aged and elderly groups, with 41.3% aged 41-60 years and 41% aged 61-80 years. Males comprised 60.3% of the cohort, and nonsmokers were the largest group at 52.8%, followed by reformed smokers (31.8%) and current smokers (15.4%).

Table 4 presents the number of medical thoracoscopies performed per year, with a relatively even distribution across the five-year period (range 17.4-23.6%).

**Table 4:** Number of thoracoscopies per year

Year	Number of thoracoscopies	Percentage (%)
1st	53	17.4
2nd	65	21.3
3rd	62	20.3
4th	53	17.4
5th	72	23.6

Tables 5, 6 detail the side and size of pleural effusion, respectively, among the study subjects.

**Table 5:** Side distribution of the study subjects

Side of pleural effusion	Number of subjects	Percentage (%)
Right	166	54.4
Left	139	45.6

**Table 6:** Distribution of study subjects based on the size of pleural effusion

Size of pleural effusion	Number of subjects	Percentage (%)
Mild	26	8.5
Moderate	96	31.5
Massive	183	60

Right-sided effusions were slightly more common (54.4%) than left-sided (45.6%). Massive effusions predominated (60%), followed by moderate (31.5%) and mild (8.5%).

**Figure 1:**
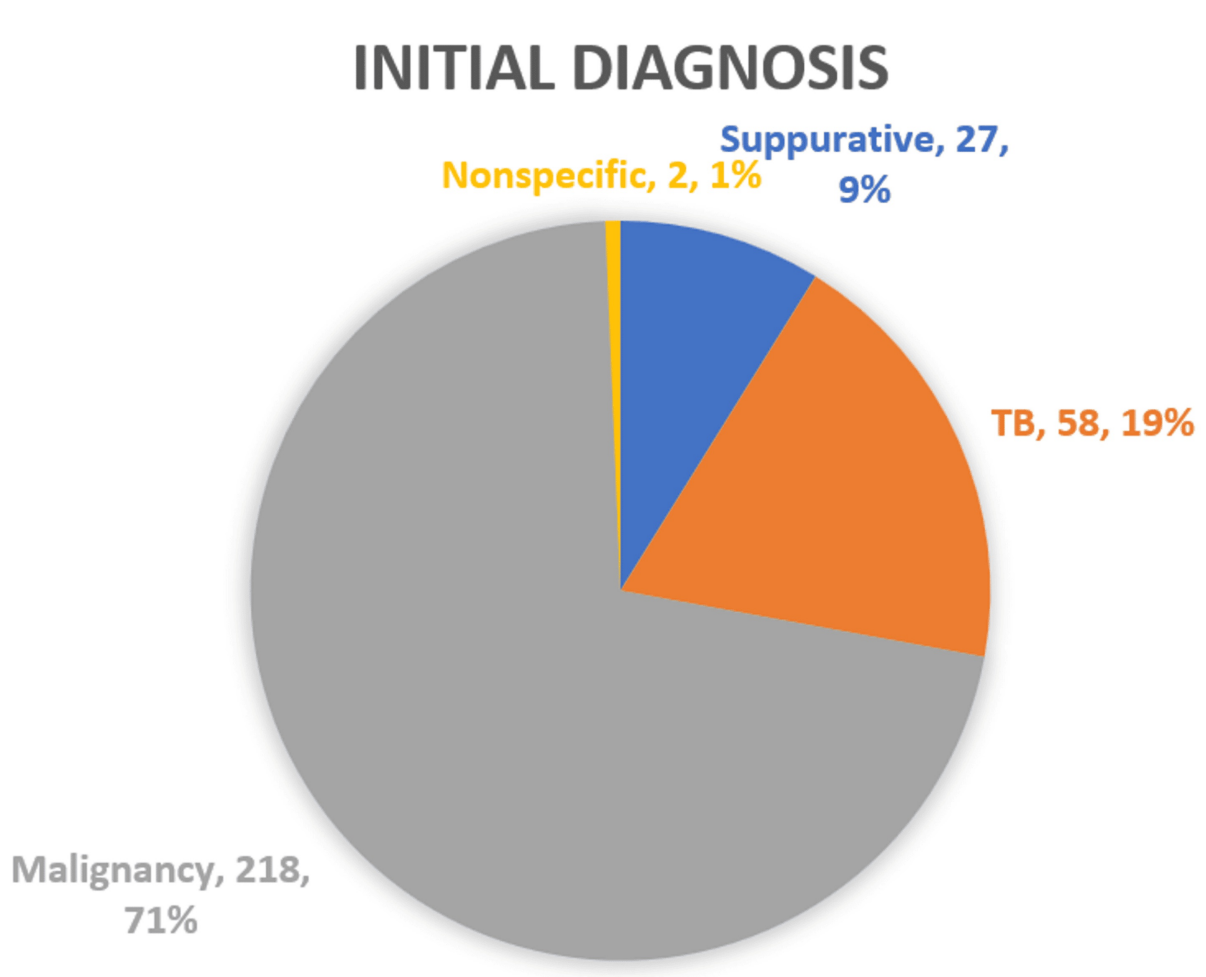
Initial diagnosis of the study subjects

The initial diagnoses of the study subjects prior to medical thoracoscopies are illustrated in Figure 1.

The findings on gross thoracoscopic examination of the pleura included adhesions, fibrinous peel, nodules, plaques, masses, inflammation/congestion, and nonspecific changes. Table 7 shows the distribution of the gross thoracoscopic appearance findings of the pleura.

**Table 7:** Distribution of the gross thoracoscopic appearance findings of the pleura

Gross thoracoscopic appearance findings of the pleura	Number of patients	Percentage (%)
Adhesions	126	41.3
Fibrinous peel	16	5.2
Nodules	182	59.7
Plaques	57	18.7
Masses	5	1.6
Inflamed/congested	58	19
Nonspecific	1	0.3
None	3	1

The most common finding on the gross macroscopic examination of the pleura was nodules, seen in 182 patients (59.7%), followed by adhesions (41.3%). The costal, diaphragmatic, mediastinal, and visceral pleura were involved in 294 (96.4%), 175 (57.4%), 48 (15.7%), and 153 (50.2%) patients, respectively.

An etiological diagnosis was obtained in 303 patients, accounting for a diagnostic yield of 99.4%. The most common histological diagnosis was malignancy, accounting for 197 (64.6%) of cases, whereas benign diagnoses accounted for only 108 (35.4%). Table 8 shows the distribution of benign diagnostic outcomes, with tuberculosis being the most common (40.7% of benign cases).

**Table 8:** Distribution of benign diagnostic outcomes

Cause of benign pleural effusion	Number of subjects	Percentage (%)
Tuberculosis	44	40.7
Suppurative	23	21.3
Nonspecific pleuritis	25	23.1
Normal	16	14.8

The distribution of malignant causes of pleural effusion is detailed in Table 9, where lung adenocarcinoma predominated (77.1% of malignant cases), followed by metastases from various sites and other primary malignancies.

**Table 9:** Distribution of malignant diagnostic outcomes

Cause of malignant pleural effusion	Number of subjects	Percentage (%)
Adenocarcinoma lung	152	77.1
Squamous cell carcinoma of the lung	11	5.6
Small cell carcinoma lung	3	1.5
Spindle cell neoplasm of the lung	1	0.5
Mesothelioma	5	2.5
Malignant melanoma	1	0.5
Non-Hodgkin’s lymphoma	4	2
Metastases from the salivary gland	2	1
Metastases from the oropharynx	1	0.5
Metastases from the ovary	4	2
Metastases from the breast	9	4.6
Metastases from the parotid	1	0.5
Metastases from the kidney	1	0.5
Metastases from the cervix	1	0.5
Metastases from the stomach	1	0.5

The immediate complications of medical thoracoscopic biopsy during and after the procedure are summarized in Table 10.

**Table 10:** Complications of medical thoracoscopic biopsy REPE: Re-expansion pulmonary edema

Complications	Number of subjects	Percentage (%)
Hemorrhage	1	0.3
Infection	13	4.5
Bronchopleural fistula	12	3.4
Subcutaneous emphysema	22	7.2
Pain	203	66.6
Fever	17	5.6
REPE	4	1.3
Cardiac complications	9	3
Death	2	0.7

The most common complication was pain, seen in 203 (66.6%) patients, followed by subcutaneous emphysema, seen in 22 (7.2%) patients that resolved spontaneously. Post-procedure fever occurred in 17 patients (5.6%) and was significantly more common in benign cases (p=0.003). Other complications were infrequent, including infection (4.5%), cardiac issues (3.0%), and bronchopleural fistula (3.4%).

Grade 1 air leaks were most commonly observed, according to the Cerfolio classification. The mean duration of air leak (bronchopleural fistula) was five days (SD ± 6). A bronchopleural fistula persisting for more than five days was considered a prolonged air leak. A prolonged air leak was observed in six (<2%) patients. In the remaining patients, the air leak gradually resolved. Eight patients developed trapped lung that required Heimlich valves. Thoracic surgical intervention was not required in any patient.

There were no complications related to sedation and/or anesthesia. Four patients developed refractory cough and dyspnea in the immediate post-procedure period that improved with high-flow oxygen and IV diuretics. None of the patients developed cutaneous infection at the entry site.

The mean duration of intercostal drainage was 10 days (SD ± 5.583). The mean duration of hospitalization was 12 days (SD ± 5.711). Two (0.7%) patients died in the post-procedure period, though none died in the immediate post-procedure period. The median time to death was 15 days (SD ± 7.8).

**Figure 2:**
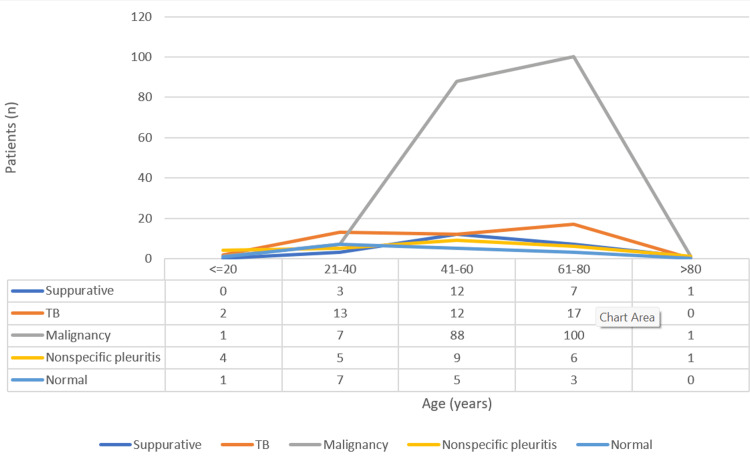
Age-wise distribution of patients according to histopathological diagnosis

Figure 2 displays the age distribution of patients by histopathological diagnosis.

**Figure 3:**
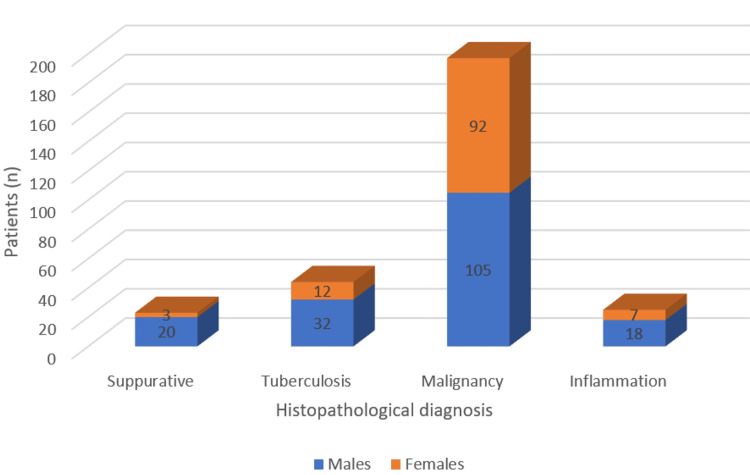
Gender distribution among suppurative, tuberculosis, malignancy, and inflammation cases

**Figure 4:**
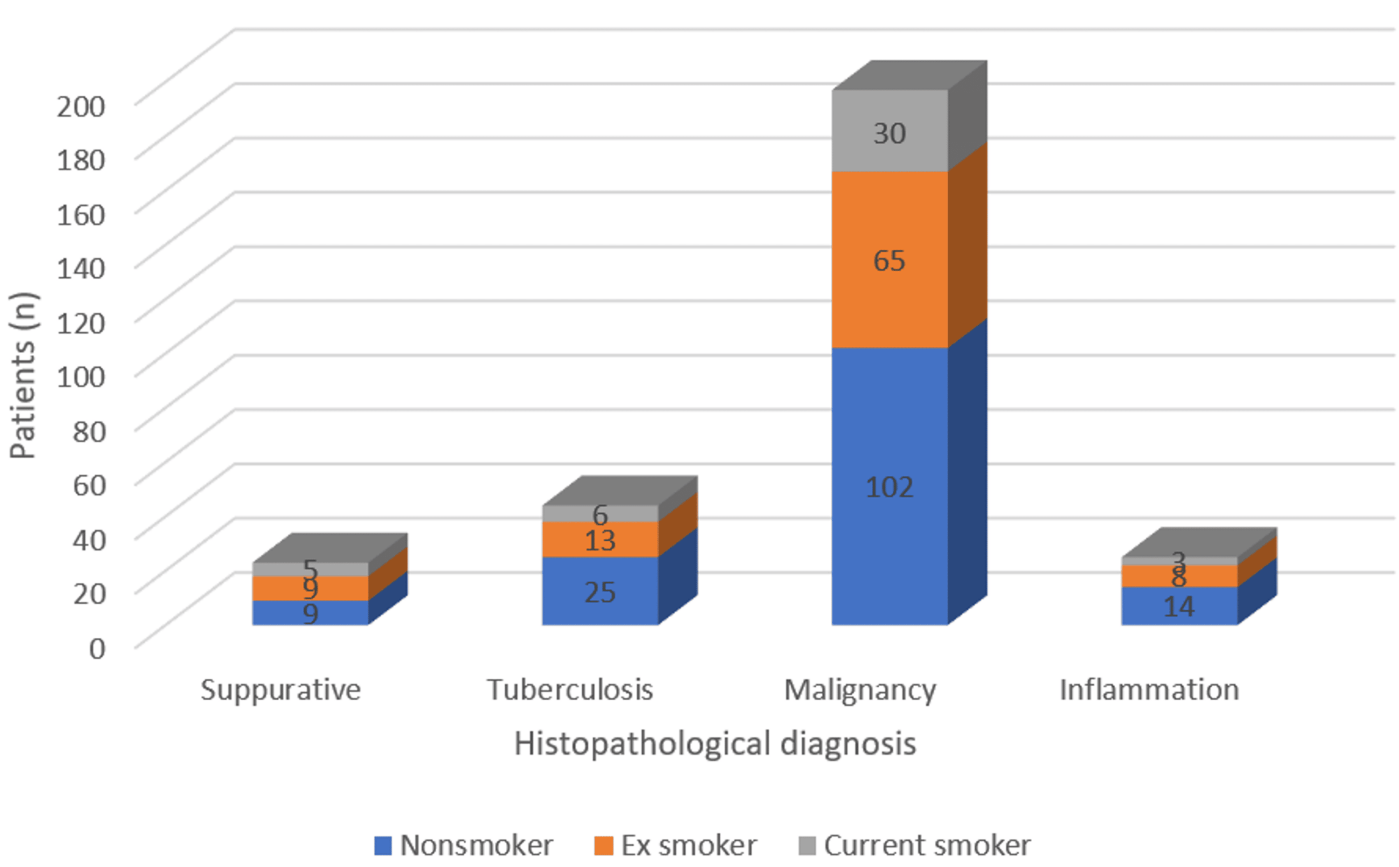
Distribution of smoking status among suppurative, tuberculosis, malignancy, and inflammation cases

The proportion of gender and smoking status among malignant, tuberculous, inflammation, and suppurative cases is depicted in Figures 3, 4.

Malignancy was significantly more common in males than females (p=0.002) and in older age groups (p<0.001). Smoking status was not significantly associated with histopathological diagnosis (p=0.679).

The distribution of histopathological diagnoses according to the gross thoracoscopic macroscopic appearance of the pleura is presented in Table 11. Nodules (59.7%) and adhesions (41.3%) were the most frequent findings. Adhesions were significantly more common in suppurative and tuberculous effusions than in malignant cases (p<0.001).

**Table 11:** Distribution of cases according to thoracoscopic macroscopic appearance

Macroscopic findings	n (%)	Suppurative (n)	Tuberculosis (n)	Malignancy (n)	Inflammation (n)	None (n)
Adhesions	126 (41.3)	16	29	57	15	9
Fibrinous peel	16 (5.2)	1	2	11	2	0
Nodules	182 (59.7)	4	21	144	11	2
Plaques	57 (18.7)	1	3	46	6	1
Masses	5 (1.6)	0	0	5	0	0
None	3 (1)	1	1	1	0	0

Chemical pleurodesis was done in 57 (18.7%) patients; 70 (23%) patients underwent adhesiolysis.

In patients with empyema, five patients required subsequent intrapleural fibrinolytic therapy for complete drainage of the pleural space. Klebsiella was isolated in only one patient. Two patients subsequently required decortication for organized empyema. Additional benign causes of exudative pleural effusion included chronic calcific pancreatitis in two patients and aortic arch aneurysm leading to pleural effusion in one patient.

The correlation between the initial clinical diagnosis and the final histopathological diagnosis obtained from the medical thoracoscopic biopsy is presented in Table 12.

**Table 12:** Correlation between initial diagnosis and histopathological result

Initial diagnosis	Histopathological result			
	Suppurative	Tuberculosis	Malignancy	Inflammation
Suppurative	15	2	2	6
Tuberculosis	6	29	6	10
Malignancy	2	13	188	9
Inflammation	0	0	1	0

There was a highly significant correlation between the presumed initial clinical diagnosis and the final histopathological diagnosis (χ2 = 203.57, p < 0.001). The majority of cases initially thought to be malignant were confirmed as malignancy on histopathology (188/212, 88.7%), with only minor misclassifications to tuberculosis (13 cases), inflammation (nine cases), or suppurative (two cases). Similarly, cases initially presumed to be tuberculous showed good concordance, with 29/51 (56.9%) confirmed as tuberculosis, though a significant proportion were reclassified as malignancy (six cases) or inflammation (10 cases). Initial suppurative diagnoses were frequently revised to inflammation (6/23) or tuberculosis (2/23), whereas no cases initially classified as inflammation were confirmed histopathologically. These findings indicate substantial diagnostic accuracy for malignancy and a moderate correlation for other etiologies in exudative pleural effusions of undetermined cause.

## Discussion

Medical thoracoscopy has become a cornerstone in the diagnosis of undiagnosed exudative pleural effusions. It facilitates direct visualization and targeted biopsies of the pleura [1,3,15]. This retrospective study of 305 consecutive patients over a five-year period in a tertiary care center in South India, with a high diagnostic yield of 99.4%, reinforces its efficacy in this diverse population.

The demographic profile - mean age 57 ± 15.5 years, male predominance (60.3%), and a substantial proportion of smokers or reformed smokers (47.2%) - aligns with patterns reported in series from India and other developing countries [6,22]. The predominance of massive effusions (60%) and right-sided involvement (54.4%) is consistent with prior observations and likely reflects referral bias toward complex cases failing initial non-invasive evaluation [6].

The diagnostic yield in our cohort exceeds that reported in most published studies. Meta-analyses and large series report yields ranging from 91% to 97% [9], while Indian studies typically cite 90-95% [20-24]. The near-perfect yield (99.4%) observed here may be attributable to rigorous patient selection, ultrasound-guided entry [8], and the procurement of multiple targeted biopsies from abnormal parietal pleural sites. The costal parietal pleura was involved in 96.4%, followed by diaphragmatic pleura (57.4%) and visceral pleura (50.2%). This reinforces the limitations of blind closed-needle biopsy techniques compared with thoracoscopic biopsy, except in debilitated patients and those with minimal pleural effusion [22].

Malignancy emerged as the leading etiology (64.6%), markedly higher than tuberculosis (14.4% overall, 40.7% of benign cases). This contrasts with earlier Indian reports where tuberculosis predominated (50-84%) [22,24], but parallels more recent trends reflecting rising lung cancer incidence, improved pre-thoracoscopy TB diagnosis via pleural fluid ADA and molecular testing, and referral of more complex malignant cases to tertiary centers [23,25]. Primary lung adenocarcinoma accounted for 77.1% of malignant effusions, consistent with global shifts toward adenocarcinoma histology and regional increases in tobacco-related lung cancer [26]. Metastatic effusions from the breast, ovary, and rarer sites further highlight the procedure's utility in identifying secondary malignancies.

There was a strong correlation between macroscopic thoracoscopic findings and final histopathology: nodules and plaques were seen more frequently in malignancy, while adhesions and fibrinous exudates were more frequent in suppurative and tuberculous effusions. These facilitated intraoperative decisions regarding talc pleurodesis, successfully performed in 18.7% of patients with suspected or confirmed malignancy and expandable lung.

The safety profile was favorable. No procedure-related mortality occurred, and major complications were rare (significant hemorrhage 0.3%, prolonged air leak <2%). The overall complication rate aligns with or is lower than meta-analytic estimates (major 1.5%, minor 10.5%) [9,20,21]. Post-procedure pain (66.6%) was most common but manageable; subcutaneous emphysema and transient fever were self-limiting. The low rates of bronchopleural fistula (3.9%) and absence of port-site infection or sedation-related events reflect adherence to standardized protocols and operator experience [3,15]. Mean intercostal drainage (10 days) and hospital stay (12 days) are comparable to published series [27].

Nonspecific pleuritis was diagnosed in 23.1% of benign cases, slightly lower than reported in some studies (25-35%). Without long-term follow-up, a subset may represent early malignancy, as observed in other cohorts where 8-25% of nonspecific results progress to cancer within one to two years [28,29].

This study has several limitations. It is a single-center retrospective study from a tertiary hospital in Kerala, India, which may limit generalizability due to local patient characteristics and referral patterns. There is also a risk of selection bias and incomplete documentation. Long-term follow-up was not available, so delayed complications, recurrence of effusion, and outcomes in non-specific pleuritis could not be assessed. There was no comparator arm; therefore, the value of medical thoracoscopy could not be evaluated relative to other approaches. A prospective, multicenter study with long-term follow-up and a comparator arm will help overcome these issues.

## Conclusions

In this single-center retrospective cohort of 305 patients with undiagnosed exudative pleural effusions, medical thoracoscopy demonstrated a very high diagnostic yield (99.4%) and a favorable safety profile, with pain as the most common complication (66.6%) and low rates of major adverse events.

These findings suggest that medical thoracoscopy is a valuable and effective tool for establishing etiology in undiagnosed exudative pleural effusions in a tertiary care center with experienced operators. However, the single-center, retrospective design, lack of long-term follow-up, and absence of a comparator arm are limitations of the study. Prospective, multicenter studies with a comparator arm and extended follow-up are needed.
